# The Epidermal Growth Factor Receptor (EGFR) as a means of neuro-immune cross-conversation

**DOI:** 10.1186/s12974-026-03873-5

**Published:** 2026-05-21

**Authors:** Katharina Gryksa, Maria Sibilia, Veit Rothhammer, Adrian Liston, Dietmar M. Zaiss

**Affiliations:** 1 Department of Immune Medicine, Faculty of Medicine, University of Regensburg, Regensburg, 93053 Germany; 2 Institute of Clinical Chemistry and Laboratory Medicine, University Hospital Regensburg, Regensburg, 93053 Germany; 3 Center for Cancer Research and Comprehensive Cancer Center, Medical University of Vienna, Vienna, 1090 Austria; 4 Department of Neurology, University Hospital Erlangen, Friedrich-Alexander-Universität Erlangen-Nürnberg (FAU), Erlangen, 91058 Germany; 5 Department of Pathology, University of Cambridge, Cambridge, UK; 6 Leibniz Institute for Immunotherapy (LIT), Regensburg, 93053 Germany

**Keywords:** Nervous system, Immune system, EGFR, HB-EGF, AREG, Neuro-immune crosstalk

## Abstract

The nervous and the immune system coevolved, and the crosstalk between both is critical for the maintenance of tissue homeostasis and mental health. In recent years, several examples have been revealed that the immune system influences behaviour, emotions, pain and even such fundamental needs as hunger. Reciprocally, several examples have become apparent in which neuronal innervation regulates local immune responses, wound healing and immune-mediated tissue homeostasis. Such findings demonstrate how well these two networks are interconnected with each other to sustain the body’s well-being. Nonetheless, the underlying mechanisms that orchestrate this interconnection remain poorly understood. The Epidermal Growth Factor Receptor (EGFR) signalling pathway exemplifies this connection, being involved in both neuronal development and maintenance as well as in immune regulation and immune mediated wound healing. Particularly two antagonistic and leukocyte-derived EGF-like growth factors, Amphiregulin and HB-EGF, have gained appreciation for their role in the regulation of local immune responses and the maintenance of tissue homeostasis. In this review, we highlight the role of these two leukocyte-derived growth factors in the regulation of the nervous system and their importance in the bi-directional crosstalk between the immune and nervous systems during tissue homeostasis and inflammation. Ultimately, we propose that under inflammatory conditions, these two leukocyte-derived growth factors may substitute typical neurotrophic factors in their function.

## Background

In recent years, it has increasingly become apparent that the nervous system can regulate peripheral inflammation [1], orchestrate immune mediated wound repair and tissue regeneration [2], as well as tailor immune responses against infections [3, 4]. Equally, there is evidence that immune responses influence behaviour and emotions [5, 6], pain [7] and even such fundamental needs as hunger [8]. These functions demonstrate the ability of one system to profoundly influence processes normally exclusively associated with the other. Indeed, several examples suggest a rather broad principle of a neuro-immune crosstalk at every level, within both the central nervous system (CNS) and peripheral nervous system (PNS). However, the molecular patterns of each of these neuro-immune cross-talks remain largely unresolved.

Strikingly, not only the immune system [9], but also neuronal development and homeostasis [10] seem highly dependent on Epidermal Growth Factor Receptor (EGFR) signalling. As an example of the latter, mice lacking EGFR expression showed severe neurodegeneration within various brain regions [11–13], with impaired expansion and self-renewal of neural stem cells (NSC), while cortical astrocytes revealed impaired signalling [13, 14]. Additionally, in EGFR null mice, peripheral nerve innervation was found to be disturbed and peripheral neurons within the skin showed hyperinnervation and disorganization [15]. Such findings strongly suggest that the EGFR signalling pathway critically influences the development, functioning and homeostasis of the nervous system.

Similarly, the immune system also uses the EGFR and its ligands for its functioning [9]. Especially under inflammatory conditions, leukocytes express two antagonistic EGFR ligands, Amphiregulin (AREG) and Heparin-Binding EGF-like Growth Factor (HB-EGF). These EGFR ligands bind the receptor with different affinities and can have antagonistic effects. Therefore, it is tempting to speculate that these two EGF-like molecules might constitute a critical mediator of neuro-immune interactions. In this review, we summarize available literature demonstrating the involvement of the EGFR signalling pathway, particularly induced by its two antagonistic ligands AREG and HB-EGF, in neuro-immune interactions within both the CNS and PNS. Furthermore, we propose that under inflammatory conditions, these two immune cell-derived EGF-like growth factors may partially substitute other neurotropic factors in their function. Such a substitution under inflammation may have substantial implications for the treatment of neuronal diseases.

### Main text

#### Amphiregulin (AREG) and HB-EGF signalling and functioning

**Fig. 1:**
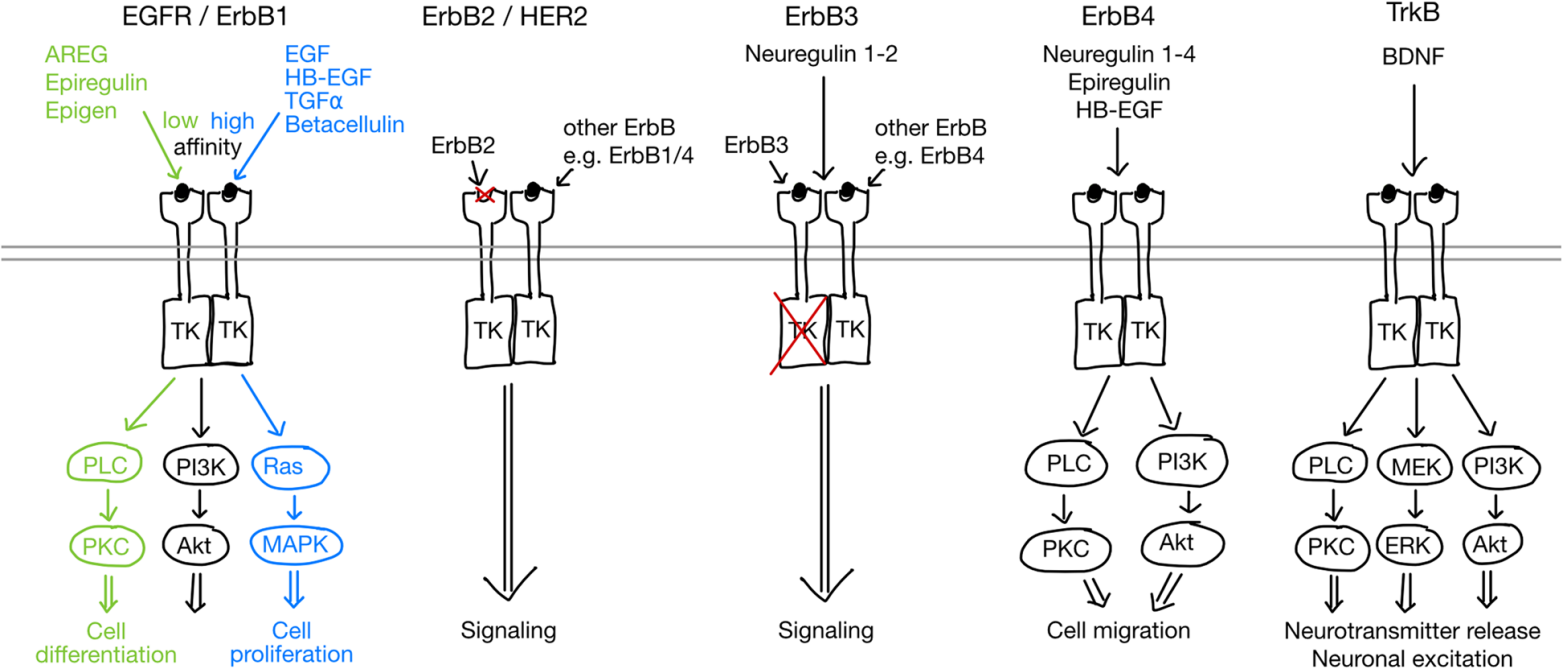
Schematic overview of EGFR family Receptor Tyrosine Kinases including ErbB1-4 and TrkB. Amphiregulin (AREG), epiregulin or epigen – which are low-affinity ligands—and epidermal growth factor (EGF), Heparin-Binding EGF-like Growth Factor (HB-EGF), Transforming Growth Factor α (TGFα) or betacellulin – which are high-affinity ligands—bind to the EGF receptor (EGFR; also ErbB1) inducing either PLC/PKC, PLC/PI3K or Ras/MAPK signalling leading to either cell differentiation or proliferation, depending on their binding affinity. ErB2 (aka HER2) with no known associated ligand, and ErbB3 with no intracellular domain, bind other ErbB to induce signalling, which is dependent on the ErbB interaction parter. ErbB3 and ErbB4 can bind neuregulin with the latter binding also epiregulin and HB-EGF leading to the activation or PLC/PKC or PI3K/Akt to affect cell migration. TrkB is also a RTK that binds the Brain-Derived Neurotrophic Factor (BDNF), affecting various signalling cascades including PLC/PKC, MEK/EGF and PI3K/Akt to modulate neurotransmitter release and neuronal excitation

Typically, EGF ligands are expressed as transmembrane proteins and become activated as they are cleaved off from the cell surface by metalloprotease, such as TACE/ADAM17. However, in leukocytes, two EGF-like growth factors AREG and HB-EGF are induced on a transcriptional level and TACE/ADAM17 readily cleaves off the translated proteins as they reach the cell surface. AREG binds the EGFR with low affinity, selectively phosphorylating the Tyrosine residue 992 (EGFR-Y992) and preferentially activates the PLC/PKC pathway (see Fig. 1) [16, 17]. As known from proteomics or transcriptomics data bases, hardly any AREG is detected within tissues under steady-state conditions [18, 19]. However, various immune cells, including macrophages, mast cells, basophils, group 2 innate lymphoid cells (ILC2), tissue-resident regulatory T cells (Tregs) and Th2 cells express AREG upon activation [9, 20, 21]. As an example, AREG expression is induced by damage-associated molecular patterns like IL-33 [22] and ATP [20], which are released following necrotic cell death or by metabolites released following apoptotic cell death [23]. In line, AREG gene-deficient mice show few abnormalities under homeostatic conditions but demonstrate impaired ability to deal with inflammatory challenges, as can be seen following helminth infection, tissue inflammation and injuries [9, 20, 21, 24–31].

AREG induces signalling via the PLC/PKC mediated “inside-out” activation of Integrin-α_V_, a known process, where PLC signalling drives cytoskeletal rearrangements within the cell, pulling the integrin subunits apart to promote a conformational opening of the integrin on the cell surface [32]. Following stimulation, e.g. tissue damage, macrophages produce AREG, which activate Integrin-α_V_ on pericytes leading to the activation of latent TGFβ. This drives the differentiation of pericytes into myofibroblasts, contributing to wound healing [20]. Thus, the ability of AREG to activate Integrin-α_V_ and subsequently TGFβ provides a wide variety of potential downstream responses from an AREG induction event, as TGFβ is known to play an important role in diverse processes, including immune regulation, wound repair, and neuronal survival [33, 34]. The AREG-TGFβ axis therefore has potential neuroimmune crosstalk functions. Under basal conditions, only low levels of AREG expression can be detected within the brain [18, 19]. However, several cell types within the brain have been described to be able to express AREG upon stimulation [35, 36], such as neurons [37] as well as glia cells, including microglia [36] and astrocytes [38], and immune cells like brain-resident Tregs [39–41], suggesting that these cells may act individually or in a synergistic fashion to influence neuronal and immunological behaviour in the brain, via amplifying Integrin-α_V_-mediated local activation of latent TGFβ. This circuit has potential to be amplified under neuroinflammatory conditions, where the primary AREG-producing cells can be increased in number or activation status.

Another EGFR ligand, shown to play a critical role for neuro-immune interactions, is HB-EGF [42]. HB-EGF is expressed under pro-inflammatory conditions, driven by exposure to TNF-alpha [43, 44]. In contrast to AREG, HB-EGF binds the EGFR with high affinity and signals mainly via EGFR-Y1068, leading to the activation of the MAPK pathway (see Fig. 1) [45, 46]. HB-EGF is a well-known mitogen for several cell types [47–49] and is itself expressed by various immune cells, including CD4^+^ T-cells, ILC3 and monocytes [44, 47, 50]. ILC3-derived HB-EGF has been found to play an important role in protecting the intestinal epithelium [44], supporting repopulation of epithelial cells and fibroblasts during wound healing [51]. Furthermore, in human and mice, glia cell-derived TGF-α and HB-EGF (see below) have been found to play key roles during recovery from multiple sclerosis (MS) or its rodent model experimental autoimmune encephalomyelitis (EAE), by decreasing axonal damage and neuronal loss [52, 53]. In this way the HB-EGF and TGF-α may represent another EGF-based axis playing a tissue-protective role in acute inflammatory lesions in the CNS [53]. As the leukocyte-derived ligands AREG and HB-EGF bind EGFR with highly dissimilar affinities, competing for EGFR binding and activating distinct signalling pathways down-stream of the receptor, these two axes of neuroimmune interaction may lead to potentially divergent physiological responses, across both systems.

#### The molecular overlap of the receptor-ligand interactions of the EGFR system

The EGFR is a Receptor Tyrosine Kinase (RTK) that belongs to the ErbB family and is broadly expressed, both by hemopoietic and non-hemopoietic cell types [9, 18, 54]. In the human genome, seven EGFR ligands are encoded, which are EGF, Transforming Growth Factor α (TGFα), AREG, HB-EGF, epiregulin, betacellulin, and epigen. In contrast to other RTK, the EGFR shows a “biased agonism” [55]. This means that upon ligand binding, the EGFR induces qualitatively distinct signal downstream of the receptor, depending on the ligand that activates the receptor [55]. High-affinity EGFR ligands, i.e. EGF, TGFα, HB-EGF and betacellulin, induced a mitogenic signal while low-affinity ligands, i.e. AREG, epiregulin and epigen, induced the differentiation of cells [55]. Upon ligand binding, EGFR activates its intracellular kinase domain, leading to the phosphorylation of specific tyrosine residues. Subsequently, intracellular signal transduction complexes then bind via their Src Homology domains to individual phosphorylated tyrosine residues and initiate a wide variety of different signalling cascades, including the PLC/PKC, PI3K/Akt and Ras/MAPK pathways [56–61].

The molecular basis of ligand differentiation for EGFR is based upon the phosphorylation of distinct tyrosine residues, activating different signal transduction pathways [57] and, consequently, having different physiological impacts on the cell [55, 57, 60]. In this way, distinct EGFR signalling can selectively modify cell functioning in antagonistic ways, by for instance inducing either cell proliferation or differentiation, or cell survival or the induction of apoptosis [9, 20, 56, 60–66]. In general, high-affinity ligands preferentially induce Ras/MAPK and/or PI3K/Akt signalling, whereas low-affinity ligands preferentially induce PLC signalling [57]. For neurons, this differential activation of EGFR mediated signalling can have critical physiological impact. For instance, in a seminal publication, the group of Phil Cohen demonstrated in in vitro studies that the high affinity EGFR ligand EGF blocked the differentiation of the neuronal precursor cell line PC12, while the Nerve Growth Factor (NGF) which binds to another RTK, the NGFR, induced neuronal differentiation [67, 68]. However, in contrast to the high-affinity ligand EGF, the low-affinity EGFR ligand AREG, induced, similarly to NGF, neuronal differentiation of PC12 cells [69]. Thus, taken together, the differential induction of specific signalling pathways downstream of the EGFR critically influences the physiological response of the signal induced.

In addition to the EGFR, the ErbB receptor tyrosine kinase family has three further members i.e. ErbB2 (also known as HER2), ErbB3 and ErbB4. Each of these receptors induces their distinct signal transduction profile. The ErbB2 has no known ligand that binds and activates it, thus, being called orphan receptor, but can interact with other RTKs, like the EGFR, ErbB3 or ErbB4, to signal. In contrast, ErbB3 can bind ligands but lacks an intracellular kinase signalling domain. Thus, ErbB3 needs to interact with other ErbB, such as EGFR, ErbB2 or ErbB4, to induce signalling. Several EGFR ligands, such as EGF, TGFα and AREG, bind exclusively to the EGFR, activating different signalling pathways depending on their affinity (see Fig. 1). But some EGFR ligands such as epiregulin and HB-EGF, can also bind to ErbB4 [70]. Consequently, the high-affinity EGFR ligand HB-EGF induces a preferentially MAPK-mediated signal via the EGFR, while the very same protein induces a PLC-mediated signal when bound to the ErbB4 (see Fig. 1) [70].

Within the nervous system, all four ErbB receptor types are expressed. ErbB1 expression is most prominent in neuronal stem and progenitor cells as well as glia cells, while ErbB2 and ErbB3 are expressed mainly in glia cells and ErbB4 in neurons and glia cells [10, 71, 72]. In addition to EGFR ligands, the ErbB receptor tyrosine kinase family can also bind neurotrophic growth factors, such as Neuregulin1 and 2, which bind to ErbB3 and ErbB4. Interestingly, EGFR has been shown to be able to directly interact with and mediate its signalling via other receptors. Prominent examples include the toll-like receptor 3, which interacts with the EGFR to modulate antiviral immunity [73], or the leptin receptor, which – as a LepR-EGFR-ERK-mediated transcytotic transporter – is essential to allow leptin entry into the brain to regulate metabolic health and food intake [74]. Furthermore, in T helper 2 (Th2) cells the EGFR interacts with the IL33R to allow T cells to function in an antigen-independent way, by inducing IL-13 expression upon exposure to IL-33 at the site of helminths infection [16].

The Tropomyosin receptor kinase (Trk) is another RTK, which binds neurotrophic growth factors including NGF (binding to TrkA), Brain-Derived Neurotrophic Factor (BDNF) (binding to TrkB), and neurotrophins 3 (binding to TrkC) and 4 (binding to TrkB) [75]. Like the EGFR, Neurotrophin/Trk signalling is involved in brain development mediating neurogenesis, neuronal proliferation, differentiation and survival, as well as neuronal morphology including axonal and dendritic growth and patterning [76–79]. However, in addition, these neurotrophic growth factors also regulate the expression and activity of various ion channels and neurotransmitter receptors as well as synaptic plasticity, which are essential for cell signalling and transduction [80]. As an example, BDNF, which is among those neurotrophins predominantly found in the adult brain, is well established to bind to TrkB in the pre-synapse and thereby increases neurotransmitter release. By contrast, BDNF binding to post-synaptic TrkB enhances glutamatergic receptor signalling and, consequently, neuronal excitation [81]. However, most interestingly, it has also been demonstrated that in the absence of BDNF, TrkB can be transactivated via EGF binding to EGFR, in this way regulating the migration of newborn cortical neurons [81, 82].

Taken together, these findings demonstrate that the EGFR signalling pathway and the signalling pathway of neurotrophic growth factors that regulate the functioning of the nervous system are closely linked to each other, and that in specific situations the function of one factor can be substituted by that of another factor.

### EGFR signalling pathway within the central nervous system (CNS)

#### EGFR signalling in the CNS

It is interesting to notice that specifically those leukocytes implicated in neuropsychiatric inflammatory connections, i.e. macrophages, ILC2, CD4 effector T-cells or Tregs, are all well-established to produce one of the two EGF-like growth factors, AREG or HB-EGF, upon activation [9, 20, 21, 44, 47, 50, 83, 84]. Thus, it is tempting to speculate that the neuroimmune crosstalk observed within the CNS could potentially be mediated, at least in part, via EGFR signalling.

In line with an EGFR-mediated neuroimmune crosstalk model, EGFR and its ligands have been repeatedly shown to be expressed within the CNS, although EGFR ligands cannot cross the blood–brain barrier [85]. They have repeatedly been shown to modulate neuronal signalling, development and neurogenesis of most cell types in the CNS, including neurons, astrocytes, oligodendrocytes and microglia [10, 86–89]. Within the hippocampus, EGFR signalling seems to regulate synaptic plasticity [90, 91] and to drive neurogenesis. For instance, injecting EGF intracerebroventricular (icv) 7 days following traumatic brain injury, significantly improved cognitive performance and reduced neuronal cell loss [92]. Furthermore, glia cell derived TGF-α and HB-EGF (see below) have been found to play key roles during recovery from MS, by decreasing neuroinflammation as well as axonal damage and neuronal loss [52, 53]. Furthermore, EGFR signalling has been implicated in schizophrenia neuropathology [93]. Here, EGF was reduced in the blood of patients compared to healthy controls [94, 95], whereas neureguin-1 was upregulated in the blood [96] and brain [97] of schizophrenia patients. In rats, intra-striatal infusion of EGF locally activated EGFR and induced behavioural abnormalities that are known in schizophrenia models [98], including low prepulse inhibition and impaired latent learning of active shock avoidance [99].

Under basal conditions, only low levels of AREG expression can be detected within the brain. Nonetheless, AREG still seems to be also involved in neural circuits regulating social dominance with increased AREG expression being correlated with an improved social rank in male mice [38]. Moreover, following cerebral ischemia, increased levels of AREG expression can be found, while experimental administration of this growth factor reduced neuronal cell death [100]. Furthermore, brain injury increased the amount of mainly Treg-derived AREG within the brain substantially, which dampened neurotoxic astrogliosis and promoted neurological recovery [39]. Like Tregs, ILC2 were shown to infiltrate the brain following injury, where they have neuroprotective effects during recovery via the release of AREG [101]. Depletion of ILC2 led to an impairment of neurological recovery [101]. Moreover, in the above-mentioned mouse model of depression, in which meningeal Tregs diminished depressive-like behaviour, the anti-depressive effect seemed to be caused by stress-induced activation of IL33-expressing astrocytes. IL-33, in turn, activate meningeal Tregs, which in turn release AREG and attenuate the depression-like symptoms [5]. Such findings strongly suggest that some critical aspects of the neuroimmune crosstalk within the CNS are mediated by AREG, affecting neurological recovery following injury and neuronal signalling at steady state, which subsequently affects behaviour.

In contrast to AREG, its antagonistic EGFR ligand HB-EGF is highly expressed within the CNS. HB-EGF expression is mainly found by astrocytes within various brain regions, predominantly hippocampus und cerebral cortex [11, 53, 102–104]. These brain regions are predominantly known for their involvement in learning, memory and consciousness [105–107]. Indeed, HB-EGF expression within the CNS was found to be protective against Alzheimer´s disease. Here, exercise-induced increased HB-EGF release was able to improve respective symptoms including impaired memory and cognition in mice. This effect could be blocked using an EGFR inhibitor, while intranasal HB-EGF enhanced memory formation [108]. Additionally, HB-EGF signalling has been shown to enhance cognitive function in a mouse model of anti-NMDAR encephalitis [109]. In agreement, conditional knockout of HB-EGF in the ventral forebrain impaired spatial memory in two different learning tasks and impaired long-term potentiation, essential for synaptic plasticity and memory formation [91]. In a mouse model of MS, astrocytic HB-EGF expression was found to be essential for the recovery from acute inflammatory lesions in CNS autoimmunity, while intranasal HB-EGF was able to attenuate neuroinflammation [53]. In rats, HB-EGF is expressed in close proximity of developing dopaminergic neurons to promote their survival, which seemed mediated via astrocytes [110]. Moreover, icv HB-EGF decreased infarct size and reduced neurological deficits, while increasing neurogenesis in a rat model of cerebral ischemia [103, 111]. Similarly, within the brainstem of newborn rats, HB-EGF had growth-promoting effects on healthy microglia, including enhanced ramification and cellular territory (reduced density but unchanged cell number) and a shift towards a complex/surveillant phenotype [112]. Also in in vitro studies, HB-EGF promoted the proliferation of CNS astrocytes, progenitor cells, and improved survival of CNS neurons [102, 104, 113]. These findings strongly show that HB-EGF is important during neurogenesis by influencing stem cell proliferation within the CNS.

Nonetheless, high concentrations of HB-EGF appeared to be not always beneficial. In mice, genetically overexpressing HB-EGF stimulated the release of vascular endothelial growth factor and was associated with hydrocephalus [114]. Furthermore, in hippocampal seizures, the HB-EGF/EGFR signalling pathway induced a switch from NSC into a reactive form, which led to impaired neurogenesis [115]. Taken together, these findings strongly suggest that a balanced EGFR signal transduction system is crucial for the function of the CNS. Furthermore, leukocyte-derived EGF-like growth factors appear to be critical mediators of the neuroimmune crosstalk within the CNS. As immunological infiltrate and activation can change the balance between EGFR ligands, the shift between immune homeostasis and neuroinflammation is likely to profoundly impact the downstream physiological processes dependent on the predominantly expressed EGFR ligand.

#### Immune cells in the brain

For a long time, it was assumed that the brain is an immune privileged organ. Indeed, the dominant immune cell type within the CNS is microglia, which are specialized, resident macrophages of the CNS with macrophage-like functions that originate from yolk sac progenitors and populate the CNS early during embryogenesis [116–119], while astrocytes, one of the most abundant cell types in the CNS, are involved in homeostatic maintenance, structural support, neuronal signalling, and metabolic regulation. Microglia represent about 10% of cells in the brain. Nonetheless, in recent years it has been increasingly appreciated that postnatal entry of leukocytes into the brain occurs, with particular representation in the barrier regions of the brain. There are multiple migration routes plausible for brain entry of leukocytes, which likely vary in importance between cell type, across developmental time and based on environmental conditions. The meningeal lymphatic vessels function as one important source of immune cells for the brain. The meninges are an immunologically active compartment that is enriched with diverse resident immune cells. Upon neuroinflammation, meningeal immune cells accumulate and may infiltrate the brain [120]. As an example, in the animal model of experimental autoimmune encephalomyelitis, meningeal inflammation has been observed prior to symptom onset and during relapse, which supports the suggestion that meninges are a gateway for immune cell access into the CNS [121]. The postnatal immune cells that become resident within the CNS have important roles in maintaining tissue homeostasis and their enrichment at border regions allows them to function as an important bridge between the periphery and the nervous systems. Changing entry of these populations has the potential to provide a peripheral immune influence over brain functions such as cognition and behaviour. Conversely, the exit of these cells, e.g. T cells, via lymphoid vessels draining the cerebrospinal fluid has the potential to carry immunological information from the brain to the periphery to regulate systemic immune responses [120, 122–125].

Within the brain, the greatest potential for information carriage by immune cells will be contained within those cells with lower residency time and higher migration rates. In this context T cells, especially CD4 T cells, such as Tregs, are important conduits of connection to the peripheral immune system. CD4 T cells have been demonstrated to regulate and control neurons and glia cells to aid proper development and communication within neurological systems [40, 126, 127]. Interestingly, inflammation within either the CNS or the PNS has repeatedly been related to behavioural changes and the development of neuropsychiatric disorders, including depression, anxiety, posttraumatic stress disorder and schizophrenia [128–131]. Such immune-neuropsychiatric connections may represent dysregulation of the immune information flowing into the brain, driving inappropriate remodelling of neurological processes. Reciprocally, and as a bidirectional phenomenon, psychiatric diseases facilitate inflammatory responses and impairs both the CNS and PNS [132]. Accordingly, clinical studies showed an increase of T helper 17 (Th17) and Th17:Treg ratio in depressed patients that were naïve to drug treatment [133]. In concordance, proinflammatory cytokines, such as IL-17, were shown to promote anxiety-like behaviour by increasing the excitability of neurons within the basolateral amygdala, while anti-inflammatory cytokines, such as IL-10, have opposite effects, acting on the same neuronal population [134]. Moreover, in a mouse model of depression, meningeal regulatory T cells (Tregs) were increased, whereas neutralization of these cells exacerbated depressive-like behaviour, indicating a protective effect of Tregs during the development of depression [5]. Finally, intravenous administration of Tregs from unstressed mice prevented the increase of stress-induced anxiety-like behaviour and the number as well as the branching of microglia in the dentate gyrus of the hippocampus when applied following chronic restraint stress [135]. Such findings strongly suggest that a pro-inflammatory state within the brain contributes to mental disorders like major depressive disorder and anxiety [133, 134].

Another well documented reciprocal neuroimmune interaction in the CNS is the experimental loss of synaptic inhibition (dis-inhibition) via reduction of Gamma-Aminobutyric Acid (GABA)ergic signalling, playing an essential role in various neuronal processes including learning and memory [136], as well as chronic pain [6, 137]. Dis-inhibition related processes seem closely regulated by immune cells, since mice that lack ILC2, a cell type predominantly located in the meninges, showed a reduction of hippocampal memory tasks, which was accompanied by a reduction of inhibitory synapses within the hippocampus [136]. However, dis-inhibition affected also inflammatory reactions in peripheral organs, such as the lung [138] and the intestine [139]. In turn, inflammation itself seems also to affect GABA transmission [138, 140], while pneumonia-induced inflammation increased neuronal activity of GABAergic neurons within the central amygdala [138], a brain region involved in emotional processes like anxiety, fear and aggression [134, 141]. Increased neuronal activity of GABAergic neurons signalling back to the lungs further enhanced the immune response, which substantially contributed to inflammation and lung injury [138].

These examples clearly demonstrate that leukocytes critically influence the functioning of the CNS on several different layers, while CNS signalling can affect peripheral immune responses.

#### EGFR signalling within the peripheral nervous system (PNS)

Not only within the CNS, but also the PNS, most cell types, including cutaneous nerves, dorsal root (DRG) and trigeminal (TG) ganglia, sensory neurons, satellite glia cells and Schwann cells express EGFR [10, 142–144]. This suggests a strong potential for impact of the EGF system on the PNS.

### Autonomic nervous system (ANS)

#### Parasympathetic (PaNS) and sympathetic (SNS) nervous system

Parasympathetic and sympathetic neurons origin from the spinal cord and brainstem to signal between the brain and the periphery. Hereby, neurons of the PaNS signal via the neurotransmitter acethylcholine (Ach), while neurons of the SNS signal via catecholamines (dopamine, adrenaline, noradrenaline) [145]. The main, reciprocal link between the ANS and the immune system is made by the *nervus vagus*, as it is their main target for communication and the longest nerve, made up by 20% efferent and 80% afferent fibers [146]. Homeostasis of the PaNS and SNS is also fundamental for tissue homeostasis. A disbalance in the ANS can lead to the development of various inflammatory pathologies like irritable bowel disease and rheumatoid arthritis, with the latter being mainly caused by low parasympathetic activity [146, 147]. Reciprocally, the pro-inflammatory cytokine IL-1β has been shown to increase the activity of sympathetic nerve innervation of visceral organs like kidney, spleen and adrenal gland [148–150].

The impact of the EGFR on the ANS is barely studied. Measuring EGF in the saliva of diabetic patients revealed increased EGF in patients with autonomic neuropathy, which is thought to be based on a hyperactive sympathetic innervation [151]. In line with such an assumption, the SNS is known to mediate EGF release, since electrical stimulation of the superior cervical sympathetic nerves led to an increased release of EGF from the submandibular gland in mice [152]. Furthermore, chemical sympathectomy using 6-hydroxydopamine prevented EGF release [153]. However, in another study, it was NGF, but not EGF, that induced parasympathetic neurite outgrowth [154], which is in agreement with a further study showing that NGF was released by neurons of the PaNS and transported anterogradely to fiber terminals, where it interacted with sympathetic axons [155].

#### Enteric nervous system (ENS)

Both parasympathetic and sympathetic neurons innervate the gastrointestinal tract acting respectively stimulating, to drive, or inhibitory, to slow down, motility, digestion and secretion.

Within the gut, gastrointestinal macrophages are known for their role in homeostasis and host defence and are in close proximity to neurons [145, 156, 157] with a high expression of the β2-adrenergic receptor. Following infection, sympathetic neurons become active and signal to macrophages via the release of noradrenaline, which in turn release polyamines to protect intestinal neurons from cell-death and pathology [158, 159]. Additionally, also other immune cells like mast cells are close to neuronal nerve endings within the ENS. Activation of mast cells leads to degranulation and thus rapid release of inflammatory mediators like histamine and serotonin, affecting sensitization of visceral pain [160, 161]. Furthermore, ILCs are resident lymphocytes that are the first responder to tissue damage and actively crosstalk with neurons within the gut [145]. Like macrophages, ILC2 express high level of the β2-adrenergic receptor and colocalize with adrenergic neurons. Thus, even 2-adrenergic signalling seems to impair type 2 immune responses and helminth host defence [145]. Nevertheless, ILC2 also express Calcitonin Gene-Related Peptide (CGRP) receptors. Of note, CGRP is released from enteric and sensory neurons, which mediate auto-inhibition of IL-13 production, acting probably as a negative feedback loop on ILC2 regulation [145, 162].

Within the gastrointestinal tract, EGF is also known as a mucosal protective factor, promoting intestinal development, barrier formation and reducing oxidative stress [65]. Even though, the EGFR ligand AREG is known to be essential for intestinal regeneration and epithelial growth following radiation injury [163], less is known about its impact on enteric neurons and neuro-immune interactions. However, since AREG promotes tissue-protection in the epithelium of the intestine [9], while macrophages, mast cells and ILC2, which highly interact with enteric neurons (see above), are a main source of AREG [9, 164], it appears highly likely that AREG might also promote neuronal homeostasis and neuronal repair within the ENS.

For HB-EGF, there are several studies showing a direct impact on neuro-immune interactions within the gut. For instance, HB-EGF promotes enteric NSC differentiation into mature neurons and induces the release of neuronal nitric oxide synthase, a factor that is reduced following neonatal necrotizing enterocolitis. Thus, HB-EGF does not only act as a mitogen for enteric NSC but also protect from neonatal necrotizing enterocolitis-induced injury [165]. Similarly, another study demonstrated that HB-EGF is essential for the development of the ENS, as it stimulates the migration of enteric neural crest cells into the gut during embryonic development [166].

#### Role of EGFR mediated signalling within the somatic nervous system

Somatosensory neurons, mainly originating from the DRG and TG, sense and mediate external and/or environmental stimuli, including touch, thermoception, thermoregulation, proprioception, itch and pain and transmit the information to the brain [167]. Sensory, especially nociceptive, neurons densely innervate the skin, where they secrete neuropeptides, such as CGRP, as well as neurotransmitters like catecholamines and GABA, modulating immune cell functioning [167–169]. Vice versa, these neurons express receptors for bacterial components, including Toll-like receptor 4, which detect lipopolysaccharides or fungal pathogens, and affect the immune response [168, 170, 171]. As an example, Transient Receptor Potential Vanilloid 1 (TRPV1) -expressing nociceptive neurons can detect different *Streptococcus* pathogens, e.g. via direct interaction with secreted streptolysin S, resulting in CGRP release, which inhibits the recruitment of neutrophils [172, 173]. Thus, immune cells and their derived signalling molecules can modulate nociceptive signalling via, e.g., TRP channels, purinergic P2X channels, mechanosensitive ion channels, G-protein-coupled receptors, as well as cytokine receptors [168], affecting neuronal excitation and pain transduction following inflammation [174]. Likewise, Tregs were found in close proximity to sensory neurons, while depletion of Tregs increased not only inflammation but also neuronal activity and subsequently pain sensitivity in male and female mice [7]. Similarly, ILC2 are important to regulate nociceptive sensitivity [175]. Depletion of ILC2 induced hypersensitivity towards mechanical and thermal pain, as well as induced gait deficits in mice [175].

During injury, nerve endings of nociceptive neurons grow into injured tissue and release those peptides that signal to immune cells to promote wound healing [176]. Interestingly, proper neuronal innervation of the skin has been found to require EGFR signalling. EGFR null mice (neonatal period) showed neuronal hyperinnervation of the skin, which were also highly disorganized, indicating disturbed neuronal outgrowth and branching of sensory neurons in EGFR null mice [15]. Another study demonstrated that following skin injury, macrophages were recruited to the sprouting axons of sensory nerves, which is mediated by neuronal-derived TGF-β [1]. Here, AREG might play an essential role, since it activates latent TGF-β [20]. In line, AREG derived from Schwann cells facilitated axon elongation, axonal regrowth and neurite outgrowth after nerve injury following sciatic nerve crush and stimulated Schwann cell migration and proliferation [2]. Fittingly, analysing DRG 12 and 24 h after sciatic nerve transection, AREG was highly expressed and ex vivo cultured DRG showed increased axonal outgrowth after AREG stimulation [177]. Thus, AREG seems to be predominantly responsible for tissue restoration following injury/inflammation, as well as neuronal outgrowth and branching.

In contrast to AREG, only little is known about the impact of HB-EGF on the somatic NS. In zebrafish, HB-EGF was upregulated following injury of the olfactory epithelium, while recombinant HB-EGF application promoted neurogenesis and neuro-regeneration of sensory neurons [178, 179]. In line, zebra fish ependymal cells of the spinal cords produce HB-EGF following injury, which has been found to be crucial for innate spinal cord regeneration and repair, whereas genetic lack of HB-EGF induced defective swim capacity and axonal repair [180].

Nonetheless, altered neuronal innervation within the somatic nervous system, especially when affected neurons are nociceptive, alters not only the immune response, but also pain perception and transduction. Since many DRG and TG-derived sensory neurons are nociceptive, it is highly likely that AREG and HB-EGF affect also pain. To our knowledge, only little is known about the involvement of the EGFR and its ligands in pain. Clinical and preclinical studies demonstrated that inhibition of the EGFR reduced, while stimulation of the same enhanced, pain sensitivity [181–184]. In oral cancer, EGFR activation has been associated with increased pain and opioid tolerance by sensitizing TG cells, while HB-EGF expression was upregulated in human oral squamous cell carcinoma [184]. In accordance, increasing the abundance of HB-EGF in a mouse model of mechanical pain enhanced pain sensitivity [185]. Unexpectedly, also AREG seems to promote neuropathic pain, since peripheral nerve injury in male and female mice increased AREG expression in DRG, whereas blockage of AREG attenuated injury-induced increase in neuropathic pain [186]. This result seems surprising since depletion of ILC2 – a main source of AREG, thus AREG should be reduced – also induced a hypersensitivity towards pain [175]. Thus, during pain behaviour, stimulation of the EGFR seems to enhance pain transduction/perception independent of ligand affinity.

## Conclusion

**Fig. 2:**
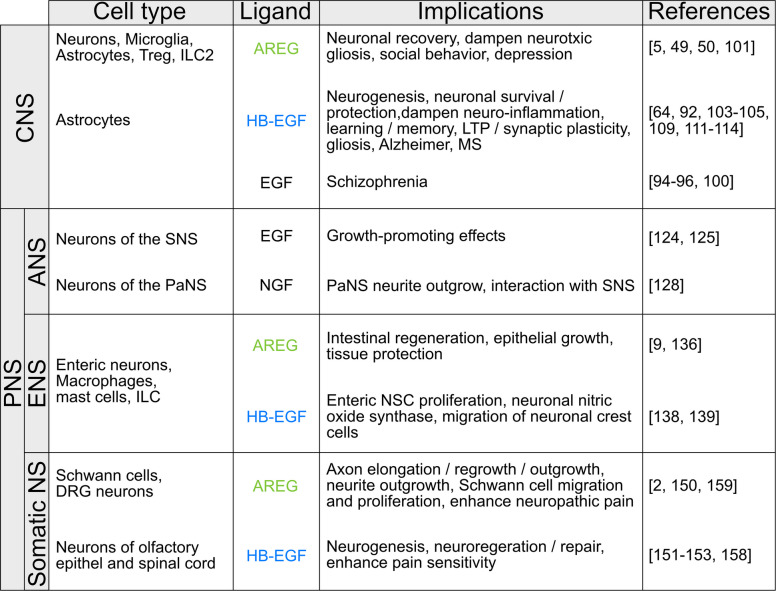
Schematic summary of cell types, ligands and implications of the EGF system within the NS. Within the central nervous system (CNS), Amphiregulin (AREG) is expressed by various cell types, including neurons, microglia, astrocytes, regulatory T cells (Treg) and Group 2 Innate Lymphoid Cells (ILC2), affecting neuronal recovery, dampening neurotoxic gliosis, and being even involved in social behaviour and depression. HB-EGF, mainly expressed by astrocytes, affects neurogenesis, neuronal survival/protection, dampens neuroinflammation and is involved in learning and memory, long-term potentiation (LTP) and synaptic plasticity, and gliosis, as well as Alzheimer´s disease and multiple sclerosis (MS). Altered EGF levels have been found in schizophrenia patients. Within the peripheral NS (PNS), less is known about the impact of AREG or HB-EGF in the autonomous NS (ANS). However, EGF and nerve growth factor (NGF) are secreted by neurons of the sympathetic (SNS) and Parasympathetic NS (PaNS), respectively. Here, EGF has growth promoting effects, while NGF stimulates PaNS neurite outgrowth and interacts with the SNS. The cell types expressing AREG and HB-EGF within the enteric NS (ENS) has not been clearly defined yet, but enteric neurons, as well as macrophages, mast cells and ILC seem highly probable. Within the ENS, AREG promotes intestinal regeneration, epithelial growth and tissue protection, while HB-EGF promotes enteric neuronal stem cell (NSC) proliferation, neuronal nitric oxide synthase, and migration of neuronal crest cells into the gut. In the somatic NS, Schwann cells and neurons of the Dorsal Root Ganglia (DRG) express AREG, promoting axon elongation, regrowth and outgrowth, as well as neurite outgrowth and Schwann cell migration and proliferation. Additionally, AREG seems to enhance neuropathic pain. Less is known about the impact of HB-EGF on the somatic NS, despite its functions in neurogenesis, neuro-regeneration and repair, as well as its pain enhancing effects

In conclusion, this review provides an overview for the role of the EGF system—particularly its antagonistic ligands AREG and HB-EGF—in neuro-immune interactions across both the CNS and PNS (see Fig. 2). For AREG, since it is expressed at very low levels at steady state, its impact on the functioning of the immune as well as the neuronal system is likely to be limited to inflammatory contexts – although its importance for neuronal survival via the local activation of latent TGFβ via integrin-αν remains to be explored. By contrast, HB-EGF is highly expressed at steady state and important for continuous cell proliferation, cell survival and tissue stability under homeostatic conditions, and is thus more likely to mediate basal neuroimmune crosstalk.

Following inflammation, expression of both ligands is strongly upregulated. Therefore, it is tempting to speculate that under inflammatory conditions, these two leukocyte-derived growth factors may substitute for typical known neurotrophic factors, like neuregulin and NGF, which are less strongly influenced by an inflammatory environment. Resembling neuregulin and NGF, AREG influences neuronal morphology and signalling, shaping synaptic processes and neuronal branching, while HB-EGF appears to play a prominent role in promoting neurogenesis. We therefore propose a model where EGFR-mediated neuro-immune crosstalk may substitute as the primary driver for functions under inflammatory conditions which otherwise are fulfilled by neurotrophic factors during embryogenesis or at steady state. This shift to an immune-dependent mechanism for these processes allows for strong modifications of neurological function during, and following, inflammation, and may be an essential part of maintaining nervous system function during acute inflammatory shocks. However, if excessively strong or prolonged neuroinflammatory conditions are initiated, such a signal substitution event may result in a pathological rewiring of neuronal pathways, contributing to neuropsychiatric, neurodegenerative or neuroinflammatory disorders. Such findings may then open new opportunities for therapies. Current examples mentioned within the text, such as the beneficial effects of HB-EGF in animal models of Alzheimer’s Disease [108] and MS [53], or AREG’s implication in depression [5] appear already promising. Thus, intranasal application of EGFR ligands like HB-EGF and AREG might provide novel treatment options also in clinical trials. Nevertheless, a better understanding of the EGFR mediated neuro-immune crosstalk during steady state or inflammation may open entirely new approaches to treat neuronal/neuroinflammatory disorders.

## Data Availability

No datasets were generated or analysed during the current study.

## Abbreviations

ANS: Autonomic nervous system
AREG: Amphiregulin
BDNF: Brain-Derived Neurotrophic Factor
CGRP: Calcitonin Gene-Related Peptide
CNS: Central nervous system
DRG: Dorsal root ganglia
EGF: Epidermal Growth Factor
EGFR: Epidermal Growth Factor Receptor
EGFR-Y992: Tyrosine residue 992 of EGFR
ENS: Enteric nervous system
GABA: Gamma-Aminobutyric Acid
HB-EGF: Heparin-Binding EGF-like Growth Factor
icv: Intracerebroventricular
IL: Interleukin
ILC2: Group 2 innate lymphoid cells
LPS: Lipopolysaccharide
LTP: Long-term potentiation
MS: Multiple Sclerosis
NGF: Nerve Growth Factor
NS: Nervous system
NSC: Neural stem cells
PaNS: Parasympathetic nervous system
PNS: Peripheral nervous system
RTK: Receptor Tyrosine Kinase
SNS: Sympathetic nervous system
TG: Trigeminal ganglia
TGF: Transforming Growth Factor
Th2: T helper 2
Th17: T helper 17
Tregs: Regulatory T-cells
Trk: Tropomyosin Receptor Kinase
TRPV1: Transient Receptor Potential Vanilloid 1
